# Pembrolizumab-Induced Hypothyroidism and Diabetes Mellitus: A Rare Case Presentation Post-Treatment

**DOI:** 10.7759/cureus.84880

**Published:** 2025-05-27

**Authors:** Jeevan Perera, Shubham Bhanot

**Affiliations:** 1 Acute Medicine, North Cumbria Integrated Care NHS foundation trust, Carlisle, GBR

**Keywords:** drug-induced hypothyroidism, endocrinopathy, ici-induced diabetes mellitus, immune checkpoint inhibitors, immune checkpoint inhibitors induced endocrinopathies, pembrolizumab, pembrolizumab induced diabetes mellitus

## Abstract

Pembrolizumab is a humanized monoclonal antibody that targets programmed death-1 (PD-1), a cell surface receptor expressed on activated T lymphocytes. By blocking the interaction between PD-1 and its ligands PD-L1 and PD-L2, pembrolizumab enhances T-cell-mediated immune responses against tumor cells. This immunotherapeutic strategy has shown significant clinical benefit in a range of malignancies, including metastatic melanoma, non-small cell lung cancer, and renal cell carcinoma.

However, its mechanism of action can also disrupt immune self-tolerance, leading to immune-related adverse events (irAEs), particularly involving endocrine organs. These irAEs may manifest as autoimmune thyroiditis, hypophysitis, or, more rarely, insulin-dependent diabetes mellitus.

In this case, the patient presented to a rural district general hospital with a several-week history of fatigue, polydipsia, and polyuria approximately two months after completing a one-year course of pembrolizumab for metastatic melanoma. Given the delayed and nonspecific symptom onset, diagnosis required a high degree of clinical suspicion. Laboratory investigations revealed severe hyperglycemia, suppressed C-peptide levels, and abnormal thyroid function tests. These findings were consistent with pembrolizumab-induced type 1 diabetes mellitus and hypothyroidism, both of which are recognized, though uncommon, endocrine irAEs.

## Introduction

Immune checkpoint inhibitors (ICIs) are a class of immunotherapeutic agents that restore and enhance T-cell-mediated antitumor responses by disrupting the interaction between inhibitory checkpoint receptors and their ligands. One such receptor, programmed cell death protein 1 (PD-1), binds to programmed death ligand 1 (PD-L1) and PD-L2, which are often upregulated on tumor cells. This interaction leads to T-cell inactivation and allows cancer cells to evade immune surveillance [1,2]. Pembrolizumab, a monoclonal antibody targeting PD-1, prevents this binding and thereby reinvigorates T-cell activity against tumor cells [1].

PD-L1 expression has been observed in various tumor types, including melanoma, non-small cell lung cancer, and renal cell carcinoma, and its expression is associated with poor prognosis due to immune escape [3]. Pembrolizumab is approved for multiple malignancies; however, immune activation may also lead to immune-related adverse events (irAEs), particularly affecting endocrine organs. A systematic review of 38 randomized clinical trials involving ICIs revealed that approximately 10% of patients developed endocrine dysfunctions, with hypothyroidism and diabetes mellitus being among the most common. Reported incidences of hypothyroidism ranged from 7% to 21%, while new-onset diabetes occurred in approximately 2.5% of patients [4]. Other less common endocrine irAEs include hypophysitis, adrenalitis, and thyroiditis.

This report describes the case of a 60-year-old male who developed both hypothyroidism and insulin-dependent diabetes mellitus two months after completing a year-long course of pembrolizumab for stage IIIb BRAF-mutated metastatic melanoma. The delayed onset highlights the importance of long-term endocrine monitoring in patients even after cessation of ICI therapy.

## Case presentation

**Figure 1:**
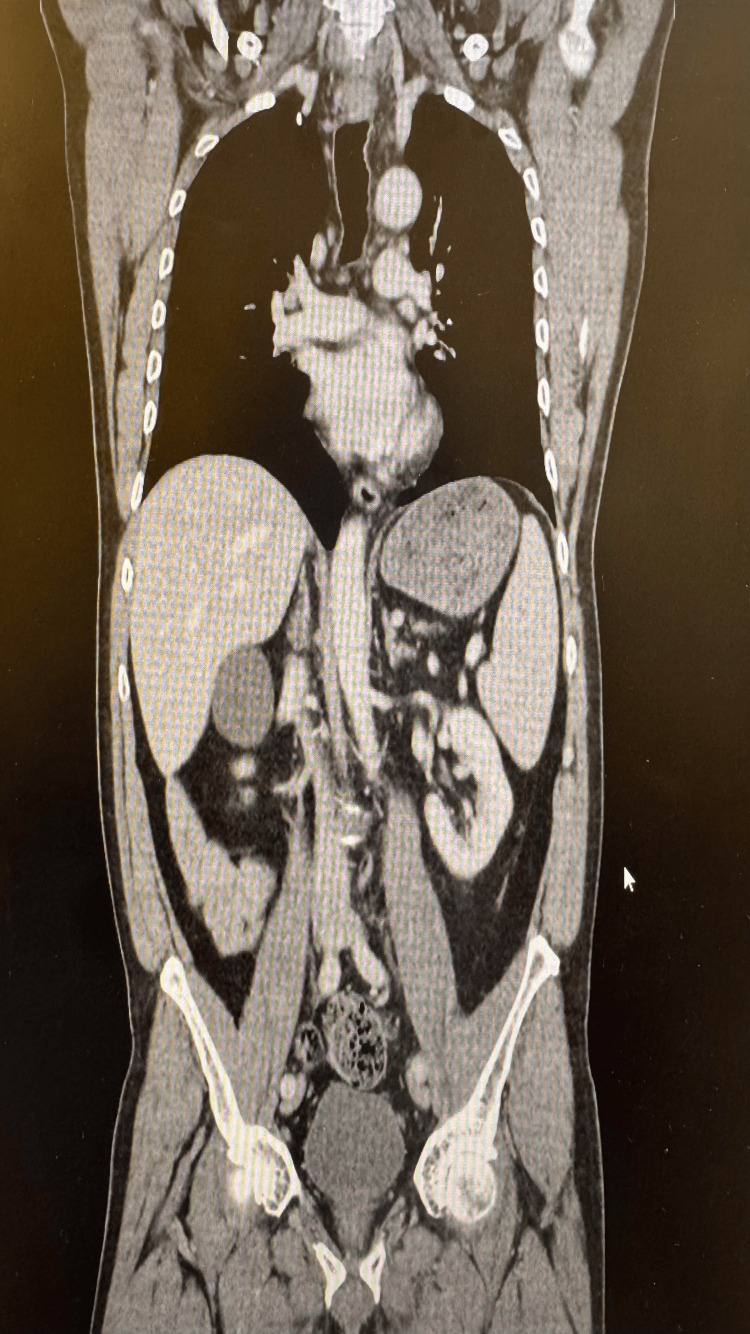
Apical view of CT imaging showing no acute abnormalities.

A 60-year-old male with a history of stage IIIb BRAF-mutated metastatic melanoma, diagnosed 14 months prior, presented with a several-week history of thirst, polyuria, and polydipsia. He had completed a 12-month course of adjuvant immunotherapy with pembrolizumab (200 mg IV every three weeks), with the final dose administered approximately eight weeks before presentation. Surveillance imaging (CT of the thorax, abdomen, and pelvis) conducted one month after therapy confirmed complete remission with no evidence of metastasis (Figure 1). He had no prior history of diabetes, thyroid dysfunction, or other endocrine disorders. He was a non-smoker and drank alcohol occasionally, with no family history of autoimmune disease.

On examination, he appeared euvolemic and afebrile, with normal vital signs. Cardiovascular, respiratory, abdominal, and neurological examinations were unremarkable. Initial laboratory investigations revealed hyperglycemia (capillary glucose 15.4 mmol/L), mildly elevated serum ketones (1.3 mmol/L), and elevated HbA1c (52 mmol/mol), suggesting new-onset diabetes mellitus. Venous blood gas showed normal pH (7.43), normal bicarbonate (22 mmol/L), and no acidosis, excluding diabetic ketoacidosis (DKA) (Table 1). Thyroid function tests showed markedly elevated thyroid-stimulating hormone (TSH 28.20 mU/L) with normal free T4 (16.0 pmol/L), suggestive of evolving autoimmune hypothyroidism. Routine blood tests (Table 2) and autoimmune screening (Table 3) were largely unremarkable: pancreatic autoantibodies (GAD, IA-2, and islet cell) were negative, and morning cortisol and parathyroid hormone were within normal limits.

**Table 1:** Investigations supporting the diagnosis of pembrolizumab-induced hypothyroidism and diabetes mellitus TSH, thyroid-stimulating hormone, HbA1c, glycated hemoglobin

Tests	Results	Reference range
Ketones	1.3mmol/L	0.1-0.6 mmol/L
Glucose	15.4 mmol/L	4.0-11.0 mmol/L
TSH	28.20 mU/L	0.30-4.50 mU/L
Free T4	16.0 pmol/L	11.0-22.0 pmol/L
pH	7.43	7.35-7.45
PCO_2_	4.2 kPa	4.6-6.0 kPa
PO_2_	6.4 kPa	11.1-14.4 kPa
std HCO_3_	22 mmol/L	22-26 mmol/L
HbA1c	52 mmol/mol	20-41 mmol/mol

**Table 2:** Results of routine blood investigations. AKI, acute kidney injury; ALT, alanine transaminase; GFR, glomerular filtration rate

	Results	Units	range
Full blood count			
Hemoglobin	175	g/L	130-180
White blood cell count	6.5	10^9^/L	4.0-11.0
Red blood cell count	5.27	10^12^/L	4.50-6.50
Platelets	178	10^9^/L	150-400
Neutrophils	4.4	10^9^/L	1.8-7.5
Urea and electrolytes			
Sodium	135	mmol/L	133-146
Potassium	4.3	mmol/L	3.5-5.3
Urea	4.5	mmol/L	2.5-7.8
Creatinine	102	umol/L	64-104
AKI stage	0		<0
Estimated GFR	69	mL/min/1.73m^2^	90-120
Calcium and albumin			
Total calcium	2.38	mmol/L	2.10-2.60
Albumin	44	g/L	35-50
Adj. calcium	2.40	mmol/L	2.10-2.60
Total protein	65	g/L	60-80
Liver function test			
Total bilirubin	16	umol/L	<21
Alkaline phosphatase	113	U/L	30-130
ALT	15	U/L	<40
C-reactive protein	<2	mg/L	<5

**Table 3:** Results of specific antibodies. PTH, parathyroid hormone

Tests	Range	References
PTH	2.4 pmol/L	1.6-6.9 pmol/L
Glutamic acid decarboxylase antibody	Negative	N/A
Islet cell antibody	Negative	N/A
Islet antigen-2 antibody	<10 IU/mL	NR 0–10 IU/mL
Cortisol	273 nmol/L	133-537 nmol/L; 6 am to 10 am

In the absence of other identifiable causes, the temporal relationship between symptom onset and recent ICI therapy strongly indicated pembrolizumab-induced endocrinopathies, specifically type 1 diabetes mellitus and primary hypothyroidism. The patient was managed on a basal-bolus insulin regimen comprising insulin glargine (10 units at bedtime) and insulin aspart (4-6 units before meals, adjusted based on pre-meal glucose readings and carbohydrate intake). Thyroid hormone replacement was initiated with levothyroxine 50 mcg once daily. He was discharged with close outpatient follow-up under the community endocrinology team.

## Discussion

ICIs such as pembrolizumab have revolutionized cancer therapy, significantly improving survival in various malignancies, particularly advanced melanoma. Pembrolizumab, a monoclonal antibody targeting the PD-1 receptor, prevents its interaction with PD-L1 and PD-L2 ligands, thereby enhancing anti-tumor immune responses [1]. Pivotal clinical trials, including KEYNOTE-001, KEYNOTE-002, and KEYNOTE-006, have demonstrated overall response rates of 33-37%, with complete responses in a smaller proportion of patients [2].

Despite these clinical benefits, ICIs are increasingly associated with irAEs, including endocrinopathies. These irAEs occur in approximately 10% of treated patients and may affect multiple endocrine organs, including the thyroid, adrenal glands, pancreas, and pituitary [3,4]. Among these, thyroid dysfunction is the most frequently observed endocrine irAE, reported in 3.9-8.5% of patients receiving PD-1/PD-L1 inhibitors [3]. It typically manifests 8-12 weeks after therapy initiation as primary hypothyroidism following a transient thyrotoxic phase. The pathophysiology is thought to involve T-cell-mediated destruction of thyroid follicular cells, potentially modulated by underlying autoimmunity and histological infiltration by CD8+ T cells and macrophages [5].

Our patient, who had no prior history of thyroid disease, developed elevated TSH levels two months following the completion of pembrolizumab. Alternative causes such as pituitary dysfunction were excluded, and the presentation aligns with reported cases of ICI-induced primary hypothyroidism, particularly those with delayed onset after treatment cessation [6].

Diabetes mellitus induced by ICIs (ICI-DM) is rare, with a reported incidence of 0.9-2% and a higher prevalence among patients receiving PD-1 inhibitors [3,7]. The clinical onset is often abrupt, presenting with significant hyperglycemia or even DKA. Mechanistically, ICI-DM results from T-cell-mediated autoimmune destruction of pancreatic β-cells. While autoantibodies (e.g., GAD, IA2) may be present, many cases are seronegative [7]. In our case, the patient exhibited marked hyperglycemia and an elevated HbA1c in the absence of DKA, and autoimmune markers were negative - features consistent with reported seronegative presentations. Notably, the patient had no previous diagnosis of diabetes, further supporting a diagnosis of pembrolizumab-associated diabetes.

While ICI-DM typically develops during treatment, delayed onset post-therapy has been observed. One review noted that 11% of ICI-DM cases occurred after treatment discontinuation [6]. This case contributes to the limited but growing body of evidence that delayed irAEs can emerge after immunotherapy has ceased [6,8].

Clinical implications

This case emphasizes the critical importance of long-term surveillance for endocrine irAEs following ICI therapy. Both hypothyroidism and diabetes mellitus in this patient emerged after pembrolizumab discontinuation, highlighting the potential for delayed toxicity. Clinicians should maintain a high index of suspicion for irAEs in ICI-treated patients, especially when nonspecific symptoms such as fatigue, weight changes, polyuria, or polydipsia arise. Prompt recognition and hormonal replacement (levothyroxine and insulin) are essential to reduce morbidity and improve outcomes.

We recommend routine monitoring of thyroid function (TSH, free T4) and glucose levels (fasting or capillary blood glucose) every 2-3 months for at least six months following ICI cessation, consistent with emerging expert consensus. Moreover, patient education is vital, informing individuals about the risk of delayed endocrine complications and the importance of early symptom reporting can facilitate timely diagnosis and intervention.

A multidisciplinary approach, including oncology, endocrinology, and primary care, enhances the management of endocrine irAEs. Proactive endocrinology involvement is particularly beneficial when patients present with unexplained metabolic or hormonal abnormalities. This case illustrates the need for prospective longitudinal studies to better define the incidence, timing, mechanisms, and reversibility of endocrine irAEs, particularly those that arise post-treatment.

## Conclusions

ICIs, such as pembrolizumab, have transformed cancer therapy but are increasingly linked to immune-related endocrine adverse events. This case highlights the development of both hypothyroidism and insulin-dependent diabetes mellitus two months after cessation of pembrolizumab, underscoring the potential for delayed onset of endocrine toxicity. Such presentations emphasize the critical need for continued vigilance and monitoring beyond the active treatment period.

Clinicians should maintain a high index of suspicion for endocrine irAEs in patients receiving ICIs, especially when nonspecific symptoms such as fatigue, weight changes, or polyuria/polydipsia occur. Early recognition and timely initiation of hormone replacement therapies can significantly reduce morbidity. Routine surveillance of thyroid and glucose parameters during and for at least six months following ICI therapy is recommended, in line with Endocrine Society guidelines.

A multidisciplinary approach involving oncology, endocrinology, and primary care is essential for the early detection and optimal management of these complex adverse events. Proactive endocrinology input should be considered at the onset of any unexplained metabolic or hormonal abnormalities. This case contributes to the growing body of evidence on ICI-induced endocrinopathies and reinforces the need for prospective longitudinal studies to better characterize the incidence, timing, and reversibility of these toxicities. Long-term follow-up and collaborative care remain central to improving outcomes in this emerging area of onco-endocrinology.
